# Establishing a prediction model of severe acute mountain sickness using machine learning of support vector machine recursive feature elimination

**DOI:** 10.1038/s41598-023-31797-0

**Published:** 2023-03-21

**Authors:** Min Yang, Yang Wu, Xing-biao Yang, Tao Liu, Ya Zhang, Yue Zhuo, Yong Luo, Nan Zhang

**Affiliations:** 1 Department of Traditional Chinese Medicine, Rheumatology Center of Integrated Medicine, The General Hospital of Western Theater Command, PLA, Chengdu, 610083 China; 2 Department of Hematology, The General Hospital of Western Theater Command, PLA, Chengdu, 610083 China

**Keywords:** Genetic markers, Genome-wide association studies, Genetics, Environmental impact

## Abstract

Severe acute mountain sickness (sAMS) can be life-threatening, but little is known about its genetic basis. The study was aimed to explore the genetic susceptibility of sAMS for the purpose of prediction, using microarray data from 112 peripheral blood mononuclear cell (PBMC) samples of 21 subjects, who were exposed to very high altitude (5260 m), low barometric pressure (406 mmHg), and hypobaric hypoxia (VLH) at various timepoints. We found that exposure to VLH activated gene expression in leukocytes, resulting in an inverted CD4/CD8 ratio that interacted with other phenotypic risk factors at the genetic level. A total of 2286 underlying risk genes were input into the support vector machine recursive feature elimination (SVM-RFE) system for machine learning, and a model with satisfactory predictive accuracy and clinical applicability was established for sAMS screening using ten featured genes with significant predictive power. Five featured genes (EPHB3, DIP2B, RHEBL1, GALNT13, and SLC8A2) were identified upstream of hypoxia- and/or inflammation-related pathways mediated by microRNAs as potential biomarkers for sAMS. The established prediction model of sAMS holds promise for clinical application as a genetic screening tool for sAMS.

## Introduction

Acute mountain sickness (AMS) is believed to be a self-limiting syndrome of nonspecific symptoms concerning fatigue, headache, nausea, and dizziness, which may occur in nonacclimatized individuals under acute exposure to high altitudes above 2500 m^1^. In certain conditions without medical care and/or in certain groups with high risks, it is possible to develop severe AMS (sAMS), sometimes even accompanied by life-threatening situations such as cerebral edema and/or pulmonary edema^2^. Generally, symptoms of mild to moderate AMS can occur early and peak within 24–72 h post high-altitude exposure, which typically overlaps the time duration when cerebral edema or pulmonary edema occurs alongside^1,3,4^. The potential continuum from AMS to sAMS, cerebral edema or pulmonary edema suggests that preventing sAMS may hold promise to avoid related events at high altitude. The occurrence of these severe disorders, to a great extent, is determined by the planned altitude, the ascent speed and the individual susceptibility; thus, the incidence of these conditions may vary greatly in different studies^3^. Accordingly, it is usually difficult to predict who is at risk of developing sAMS for prevention purposes.

Partial pressure of oxygen (PaO_2_)^5^, the partial pressure of carbon dioxide (PaCO_2_)^6^, the saturation of oxygen (SaO_2_)^7,8^, arterial oxygen content (CaO_2_)^9^, oxygen tension at 50% haemoglobin saturation (P50)^10^ and haemoglobin^11^ were thought to be hypoxia-sensitive and evidenced either as independent predictors or factors related to the subsequent development of AMS; however, speculation remains regarding their importance in the prediction of AMS. To date, studies concerning the predictive value of blood gas testing are rather limited. Blood gas findings are usually inconsistent for possible interference from field or laboratory conditions or individual reasons. Additionally, statistically significant differences usually require large-scale and/or randomized controlled trials, which are currently almost impossible to complete under high-altitude circumstances. In addition, pulmonary function testing^12^, cardiopulmonary exercise testing^13^, and hypoxic exercise testing^14^ have been used to assess the risk of hypoxemia, but the applicability of these measurements to high-altitude exposure has not been fully established^2^.

Despite the variety of the ascent plan and the individual baseline medical conditions, genetic susceptibility is addressed to explain why AMS and the related events may still occur in certain groups^15^. Genetic issues concerning AMS have been previously discussed^16–20^, however, evidence for the genetic susceptibilities to AMS is still very rare, even less to sAMS. In the study, microarray data of GSE103927^21^ were explored for the genetic background of AMS, and a prediction model of sAMS was established by machine learning of the support vector machine recursive feature elimination (SVM-RFE) method^22^, which was clinically applicable as tested within the timeline of the GSE103927 cohort and validated in an isolated cohort GSE52209^23^. Five featured genes (EPHB3, DIP2B, RHEBL1, GALNT13, and SLC8A2) were identified as important regulators of hypoxia-related processes, including erythrocyte differentiation, alpha–beta T cell differentiation, and secretion of histamine by mast cells. The study was a preliminary attempt to explore the genetic susceptibility of sAMS, which occurred in almost half of the GSE103927 subjects exposed to very high altitude (5260 m), low barometric pressure (Pb, 406 mmHg), and hypobaric hypoxia (VLH).

## Results

### Exposure to VLH activated gene expression in leukocytes

**Figure 1:**
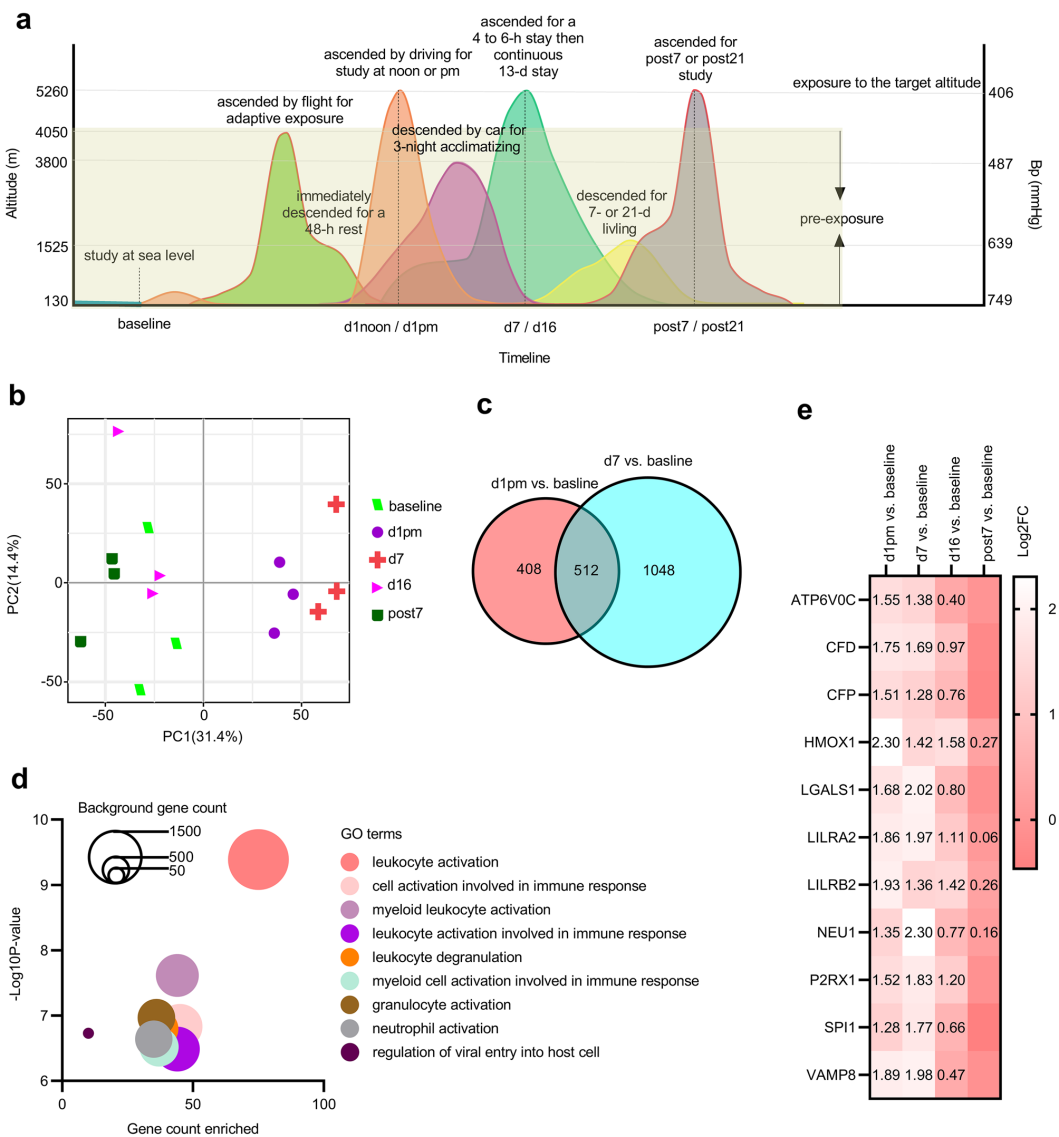
Acute exposure to VLH caused leukocyte activation. (**a**) Altitude-timeline plan of GSE103927. Subjects were studied at sea level for 30 days and then ascended to 4050 m by flight, followed by an immediate descent to 1525 m for a 48-h rest. After that, subjects ascended to the target altitude 5260 m for the d1 study at noon and pm, then decended to 3800 m for 3-night acclimatizing, allowing a 4- to 6-h exposure at the target altitude, then a continuous 13-day exposure at 5260 m for d7 and d16 studies. After that, subjects were allowed to stay at 1525 m again for 7 or 21 days and then reascended for the post7 or post21 study. (**b**) PCA for timeline gene expression patterns. (**c**) Venn analysis. (**d**) GO analysis. (**e**) Timeline changes in 12 DGs related to leukocyte activation.

The microarray data of GSE103927 were based on 112 peripheral blood mononuclear cell (PBMC) samples collected from 21 subjects at seven time points of VLH exposure. At baseline, subjects were studied at sea level (130 m, Pb = 749 mmHg). At the first day noon (d1noon) or post meridiem (d1pm), d7, d16, post-decent day 7 (post7), or post21, subjects ascended or reascended to the target altitude (5260 m, Pb = 406 mmHg). All the subjects were sampled and studied following each rapid ascending to, or prolonged stay at the target altitude (Fig. 1a). Laboratory values concerning PaO_2_ (mmHg), PaCO_2_ (mmHg), SaO_2_ (%), CaO_2_ (mL/dL), P50 (mmHg), hemoglobin (g/dL), Lake Louise Questionnaire (LLQ)-AMS score, and AMS-C-Composite score were detected and recorded. Principal component analysis (PCA) was conducted to determine the timeline differences in gene expression (Fig. 1b). Gene expression patterns at d1pm and d7 indicated an apparent distinction from baseline (increasing along the PC1 axis), but this trend was reduced along both the PC1 and PC2 axes at d16 and post7, suggesting that acute exposure to VLH may change the gene expression pattern in monocytes. Then, the differentially expressed genes (DGs) across the baseline, d1pm, and d7 were determined by *P* value (< 0.05) and Log2(fold change, FC) (> 1.3 or < − 1.3). There were 512 overlapping genes with consistent significance along the exposure timeline from d1pm to d7 (Fig. 1c, Supplementary Table S1). We then conducted gene ontology (GO) analysis to identify the function of the 512 DGs, and it was indicated that most of them were involved in leukocyte activation (Fig. 1d). Twelve DGs enriched in the GO term “leukocyte activation involved in immune response” were further explored for their expression level along the timeline (Fig. 1e). These leukocyte activation-related genes were upregulated along the timeline with a peak at d7, suggesting that leukocytes were activated upon VLH, but they recovered along the exposure timeline.

### T cells dominated the genetic responses upon VLH exposure

**Figure 2:**
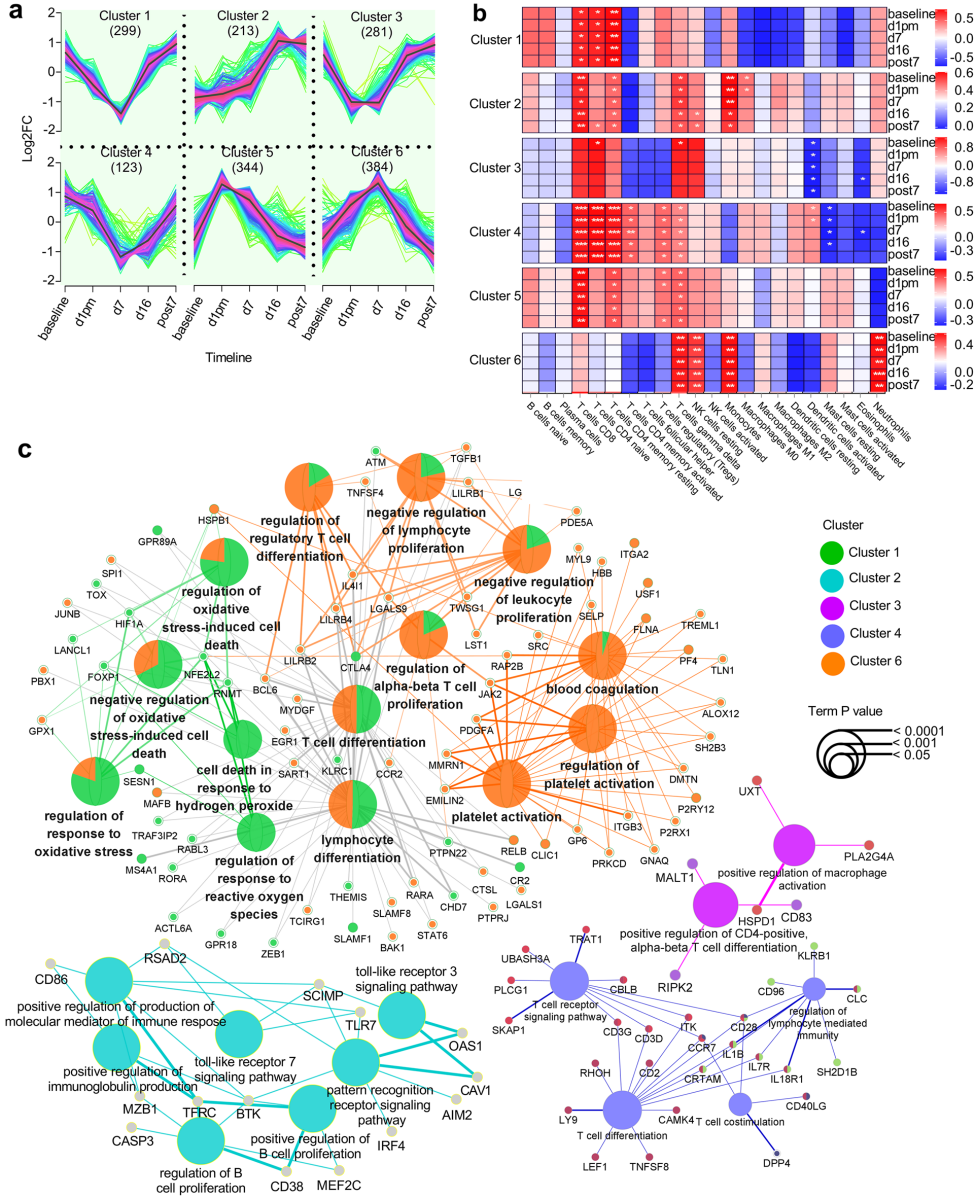
The genetic and immune functional responses to VLH exposure. (**a**) Timeline genetic responses of 1644 DGs in the six clusters. Gene expression changes were normalized using log2. (**b**) Dynamic leukocyte functional differentiation in each cluster. Backgrounded by LM22, each cluster was significantly annotated with genetic associations to different leukocyte types (two-tailed spearman correlation test, N = 22, *P < 0.05, **P < 0.01, ***P < 0.001). (**c**) Functional enrichment analysis based on the GO databases. A built-in ClueGOPlugin (v.2.5.9) in the software Cytoscape (3.9.1) was applied in the GO analysis.

To investigate the functional differentiation of leukocytes upon VLH exposure, 1644 genes (Supplementary Table S2) with significant differences from the baseline were functionally clustered along the timeline, and six clusters were obtained (Fig. 2a). Both cluster 1 (299 genes) and cluster 6 (384 genes) were inversely changed across the timeline, with a peak decrease in cluster 1 and an increase in cluster 6 at d7. Different expression dynamics were also observed in cluster 2 (213 genes), cluster 3 (281 genes), cluster 4 (123 genes), and cluster 5 (344 genes). The function of each cluster was annotated using leukocyte 22 data matrix (LM22)^24^ (Fig. 2b). As expected, each cluster was functionally associated with different leukocyte types (two-tailed spearman correlation test, N = 22, *P* < 0.05), with CD4/CD8 T cells dominating in clusters 1/2/4/5, regulatory T cells in clusters 4/5, gamma delta T cells in clusters 2/4/5/6, resting NK cells in cluster 6, monocytes in clusters 2/6, activated dendritic cells in cluster 3, and neutrophils in cluster 6. We then compared the biological functions among the clusters using the GO analysis (Fig. 2c). No significant terms were enriched in cluster 5. No relationship was observed in the expression pattern between clusters 2, 3, and 4. Both clusters 1 and 6 overlapped in T cell-associated functions, oxidative stress, and blood coagulation. Cluster 1 was specifically enriched in cell death in response to hydrogen peroxide and regulation of response to reactive oxygen species. Cluster 6 was specifically enriched in platelet activation. Both clusters 1 and 6 were indicated with dramatic altitude or hypoxia sensitivity, and both were functionally associated with T cell activities, suggesting the dominant roles of T cells in the genetic responses to VLH exposure.

### The inverted CD4/CD8 ratio may function as a risk factor for sAMS

**Figure 3:**
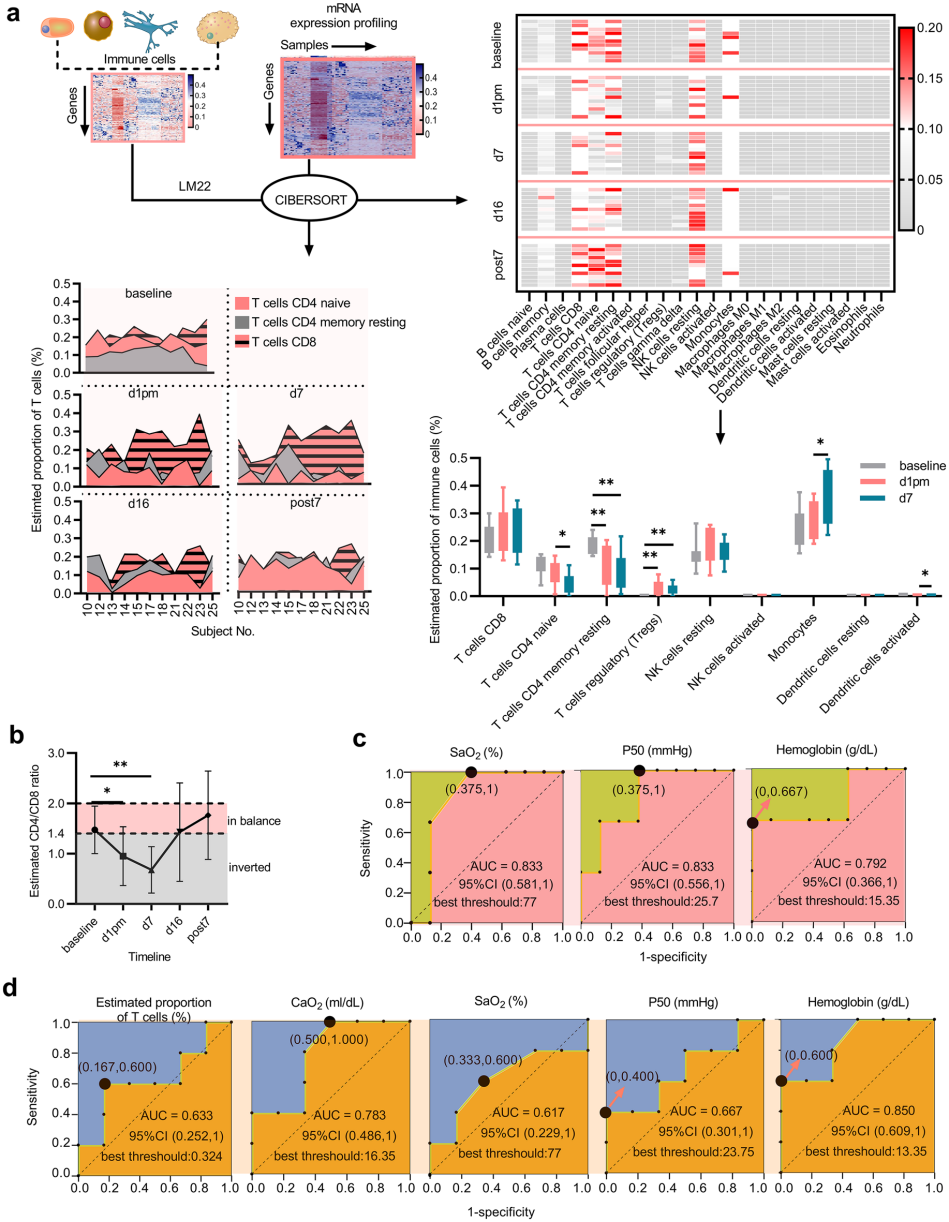
The potential risk factors for sAMS. (**a**) CIBERSORT algorithm coupled with LM22 revealed the timeline proportion of leukocytes in each subject. As indicated in the heatmap, resting T cells and NK cells were dominantly proportioned in subjects exposed to VLH. The proportion of CD4 T cells was predicted to be significantly decreased at d1pm and d7 compared with that at baseline. The bar chart indicates the changes in the average cell proportions across baseline, d1pm, and d7 (N = 11, paired t test, *P < 0.05, **P < 0.01). The error bars indicate the 95% CI. The timeline changes in CD4 and CD8 T cells in each subject in the training cohort are displayed with the area chart. (**b**) The inverted CD4/CD8 ratio. The average estimated CD4/CD8 ratio decreased from baseline to d1pm and then increased from d7 to post7 (N = 11, paired t test, *P < 0.05, **P < 0.01). (**c**) Binary classifiers of the CD4/CD8 ratio and the best thresholds. (**d**) Binary classifiers of sAMS and the best thresholds.

To investigate the timeline abundance of various leukocytes in subjects (N = 11) exposed to VLH, the cibersort algorithm^25^ was applied in combination with LM22, and expression profiling was performed at baseline, d1pm, d7, d16, and post7 (Fig. 3a and Supplementary Table S3). CD8 and CD4 T cells, resting NK cells, and monocytes were indicated as the major cell types in PBMC samples, with a significant decrease (paired t test, *P* < 0.05) in the estimated proportion of CD4 cells at d1pm and d7 vs. baseline, which resulted in significantly inverted CD4/CD8 ratio at d1pm and d7 (N = 11, paired t test, *P* < 0.05; Fig. 3b, Supplementary Table S4). When predicted using the area under the receiver operating characteristic curve (ROC AUC) (N = 11), SaO_2_ (AUC = 0.833), P50 (AUC = 0.833), and the serum level of hemogbin (AUC = 0.792) performed well in differentiating subjects with a normal or inverted CD4/CD8 ratio (Fig. 3c), suggesting their potential effects on CD4/CD8 balance. SaO_2_ (AUC = 0.617), CaO_2_ (AUC = 0.783), P50 (AUC = 0.667), hemogbin (AUC = 0.850), and the estimated proportion of T cells (AUC = 0.633) were also indicated as possible binary classifiers for sAMS (sAMS or non-sAMS) (Fig. 3d). The interplay effects between the CD4/CD8 ratio, SaO_2_, P50, hemoglobulin, the estimated proportion of T cells, and CaO_2_ implied that the inverted CD4/CD8 ratio may function as the potential risk factor for sAMS.

### Genetic profiling for sAMS

**Figure 4:**
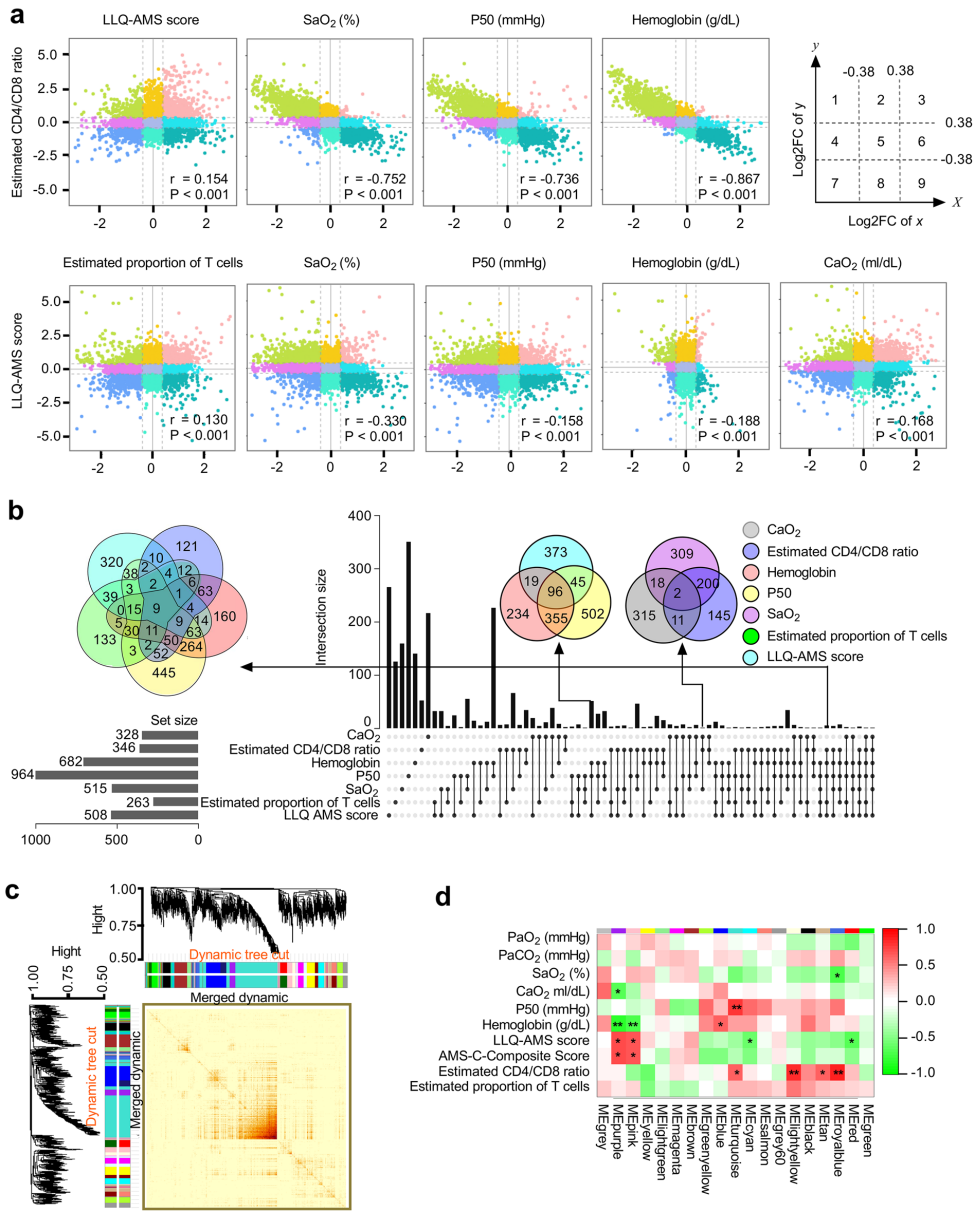
The gene signature underlying the phenotypes of sAMS. (**a**) Nine-quadrant diagram of the expression patterns of DGs across subgroups. The expression pattern was indicated with the log2 normalized FC. All DGs with log2FC > 0.38 or < -0.38 were visualized. Quadrant 1, DGs upregulated in classifier y but downregulated in x; quadrant 9, upregulated in x but downregulated in y; quadrant 3, upregulated in both x and y; quadrant 7, downregulated in both x and y. No significant correlations were observed in quadrants 2, 4, 5, 6 and 8. A total of 2286 DGs with log2FC > 1.3 or < -1.3 were selected for further analysis. (**b**) Venn analysis. The 2–6 intersections of DGs are indicated in the UpSet Venn diagram. The intersection size was displayed using upset bars and 3-way or 6-way Venn diagrams. (**c**) Network heatplot of the selected genes; (**d**) Module-trait relationships (Pearson correlation test, *P < 0.05, **P < 0.01).

To uncover the gene signature underlying the phenotypes of sAMS, subjects in the training cohort were binarily subgrouped with the best predicted thresholds of various classifiers. The interplay effects between classifiers were investigated regarding the expression pattern of DGs across the binary subgroups (Fig. 4a). Significant correlations were observed between SaO_2_, P50, hemoglobin, and the CD4/CD8 ratio in the expression pattern of related DGs (Pearson correlation test, *P* < 0.001). Meanwhile, as the potential risk factors of sAMS, the CD4/CD8 ratio, the estimated proportion of T cells, SaO_2_, P50, hemoglobin, and CaO_2_ were indicated with significant correlations with the LLQ-AMS score (Pearson correlation test, *P* < 0.001). A total of 2286 risk factor-related DGs were identified, with 328 in the set of CaO_2_, 346 in the CD4/CD8 ratio, 682 in hemoglobin, 964 in P50, 515 in SaO_2_, 263 in the estimated proportion of T cells, and 508 in the LLQ-AMS score (Supplementary Table S5), which were intersected in 2–6 ways under Venn analysis^26^ (Fig. 4b). To further identify function modules among the 2286 DGs and the relationship to the risk factors of sAMS, weighted correlation network analysis (WGCNA)^27^ was performed using the values of PaO_2_, PaCO_2_, SaO_2_, CaO_2_, P50, hemoglobin, LLQ-AMS score, AMS-C-composite score, CD4/CD8 ratio, and the estimated proportion of T cells as a trait. A gene coexpression network was constructed, and 7 modules were identified using hierarchical clustering, dynamic tree cut, and merged dynamics tools (Fig. 4c). Next, we established module-trait relationships (Fig. 4d). The royalblue module was negatively correlated with SaO_2_ but positively correlated with the CD4/CD8 ratio. Both the purple and pink modules were negatively correlated with hemoglobin but positively correlated with the LLQ-AMS score and AMS-C-composite score. Moreover, the purple module seemed to be sensitive to CaO_2_; blue, to hemoglobin; turquoise, to P50; tan, yellow and turquoise, to CD4/CD8 ratio; and red and cyan, to LLQ-AMS score. The results demonstrated that SaO_2_, CaO_2_, P50, hemoglobin, LLQ-AMS score, AMS-C-composite score, CD4/CD8 ratio, and the estimated proportion of T cells, as potential risk factors for sAMS, may impact the disease outcome at the genetic level.

### Machine learning to establish the prediction model of sAMS

**Figure 5:**
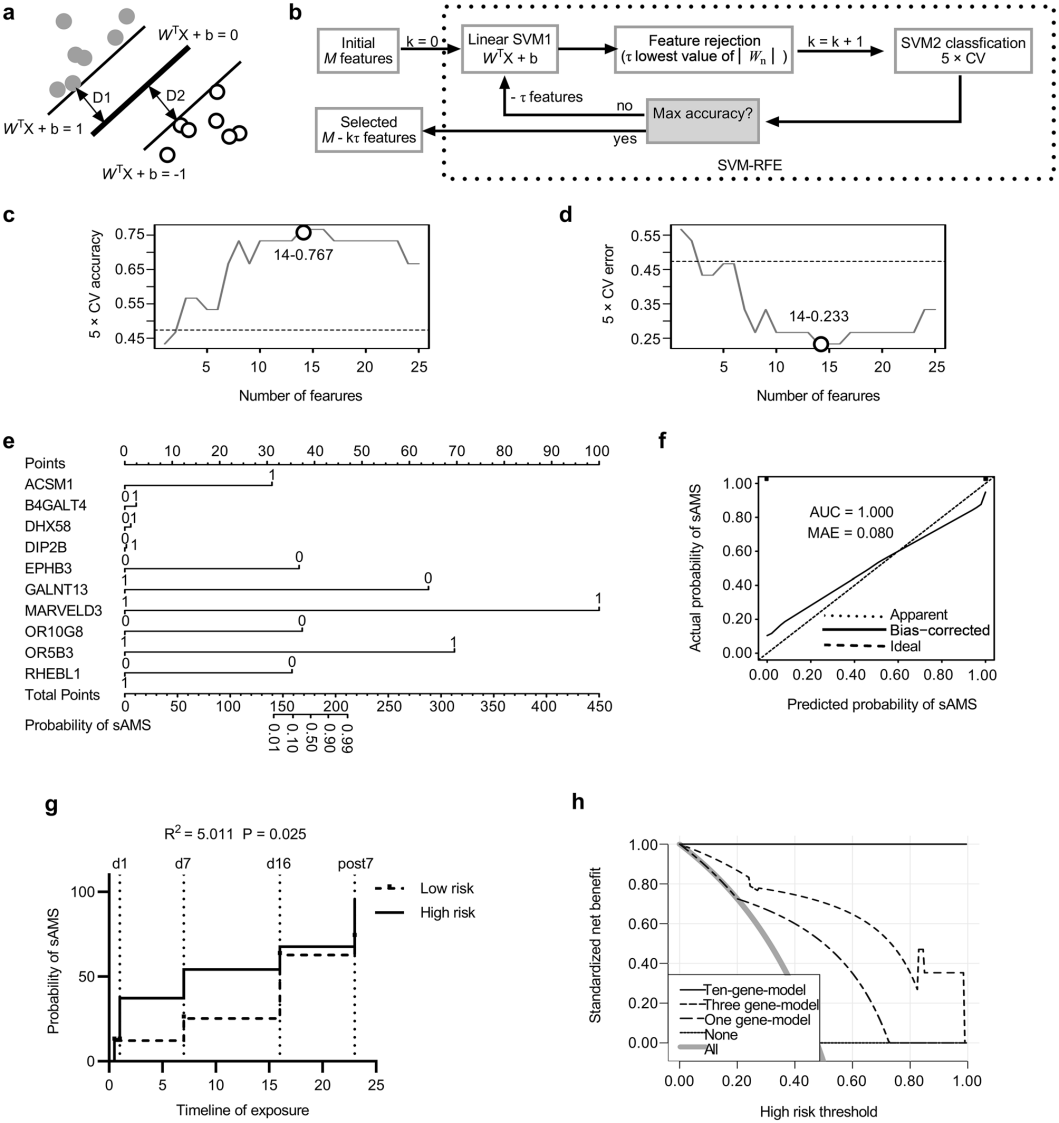
Establishing the model of genetic susceptibility to sAMS. (**a**) Hyperplane of SVM-RFE. Thicker line, hyperplane; thinner lines, margin limits; *W*^T^, the weight vector; *X*, the feature vector; b, the threshold; D1 and D2, marginal distances. (**b**) SVM-RFE method. The SVM1 parameters (absolute values of *W*_n_) were computed to determine the relevance of all the input features, and then the entire feature selection and error estimation process were performed during SVM2 classification as a guide to choose the optimal number of features. *M*, number of input features; k, number of folds of CV; τ, number of features to be rejected. (**c**,**d**) The estimated accuracy and error. (**e**) A nomogram for the prediction of sAMS upon rapid exposure to VLH. (**f**) The calibration performance of the model. (**g**) Survival analyses for the subjects in the training cohort. (**h**) DCA of the three models. The high-risk threshold was predicted as 47%.

To identify the marker genes of sAMS, we constructed a prediction model of sAMS using the machine learning of SVM-RFE, which consists of the classification algorithm and the feature selection algorithm wrapped around, strategized to select or remove some features from the high-dimensional feature set, and obtain the optimum feature subset from various candidate subsets generated. Therefore, SVM-RFE is actually designed to find a hyperplane of the maximized marginal distance with the best differentiating performance between the two categories of the dataset, which is represented with the weight vector *W*^T^, feature vector *X*, and threshold b as follows: *W*^T^*X* + b = 0 (Fig. 5a). Obviously, when *W*^T^*X* + b = 0, the sum of the marginal distances from the hyperplane to the closest features (D1 + D2) is maximized; however, it would be indicated with the poorest differentiating performance and accuracy whenever *W*^T^*X* + b = 1 or − 1. In machine learning of SVM-RFE (Fig. 5b), the initial features (*M* = 2286) were input for classifier training, with the relevance of the n-th entry of *X* determined by the corresponding value *W*_n_ in *W*^T^(n = 1, 2,…*M*). Then, in each fold (k = 15) of cross validation (CV), the concrete number of features (τ = 30) with the lowest absolute values of *W*_n_ were rejected. The maximum accuracy was determined by the entire feature selection and error estimation process (five-fold CV). The top 14 ranked features (Supplementary Table S6) with the highest five-fold CV accuracy (Fig. 5c) or the lowest error (Fig. 5d) were selected for further analysis. Ten of 14 with significant predictive power (N = 19, univariate logistic regression test, *P* < 0.05) (Supplementary Table S7) were used to build the model (C-index = 1, *P* < 0.01) (Supplementary Table S8) and nomogram (Fig. 5e). In the training cohort of d1, the model had excellent prediction performance for sAMS as analysed using ROC (AUC = 1) (Supplementary Fig. S1a), calibration curve (Fig. 5f), and survival analysis (R^2^ = 5.011, P = 0.024) (Fig. 5g). When tested using ROC within the timeline of the training cohort over baseline (N = 21, AUC = 0.600), d7 (N = 18, AUC = 0.691), d16 (N = 21, AUC = 0.673), post7 (N = 14, AUC = 0.633), and validated in the validation cohort (N = 31, AUC = 0.626) (Supplementary Fig. S1b–f), the model was indicated with satisfactory predictive accuracy between the actual probability and the predicted probability. To assess the clinical applicability of the model, we also established a single-gene (OR10G8) model (C-index = 0.764) using the baseline data of the training cohort (Supplementary Fig. S2, Supplementary Table S9) and a three-gene model (B4GALT4, DIP2B, GALNT13) (C-index = 0.897) (Supplementary Fig. S3) based on the validation cohort data (Supplementary Table S10, S11). All the models were indicated with overall net benefits varying from 53 to 100% when assessed using decision curve analysis (DCA) (Fig. 5h).

### MicroRNAs (miRs) mediated the effects of the featured genes in the development of sAMS

**Figure 6:**
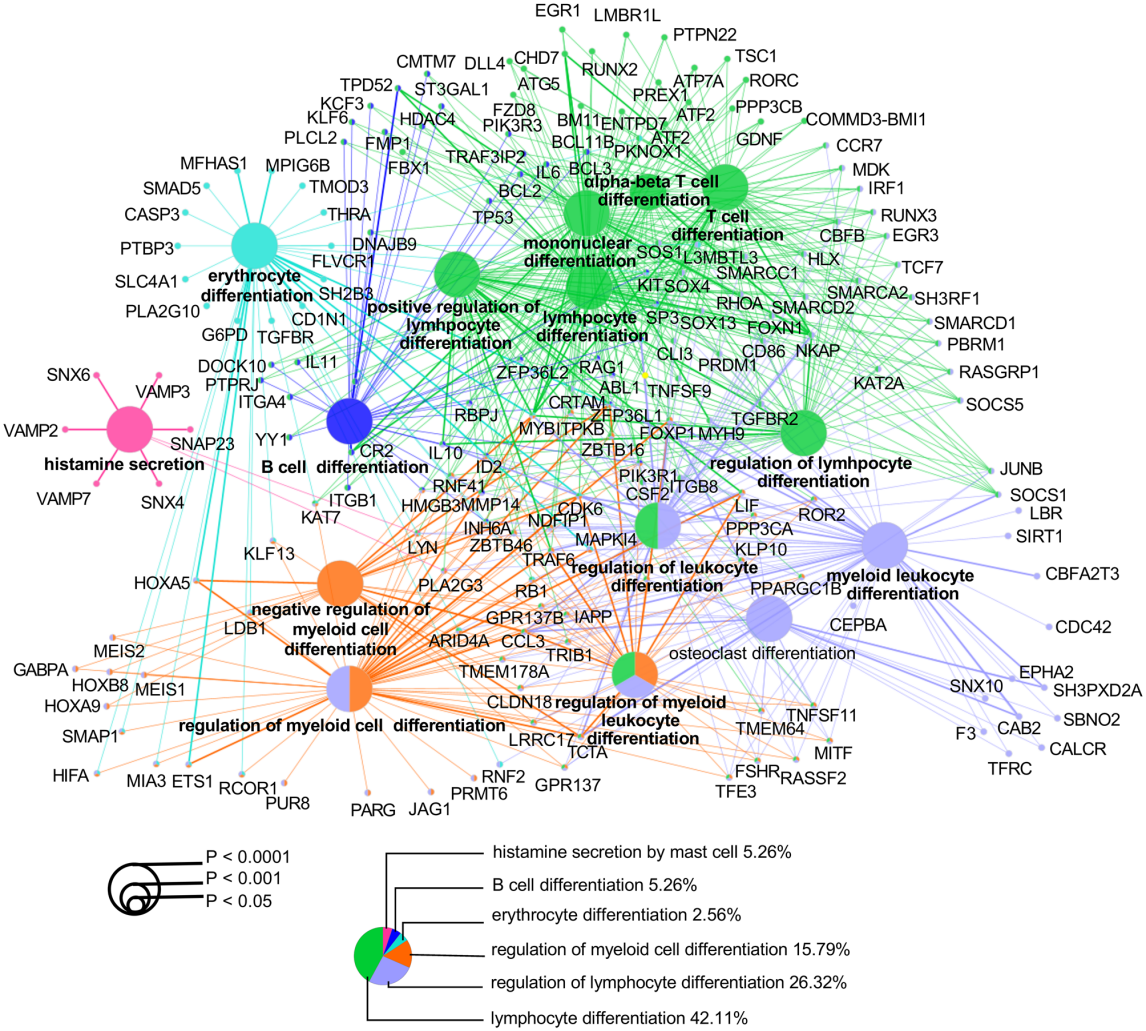
Functional enrichment of the miR targets.

**Figure 7:**
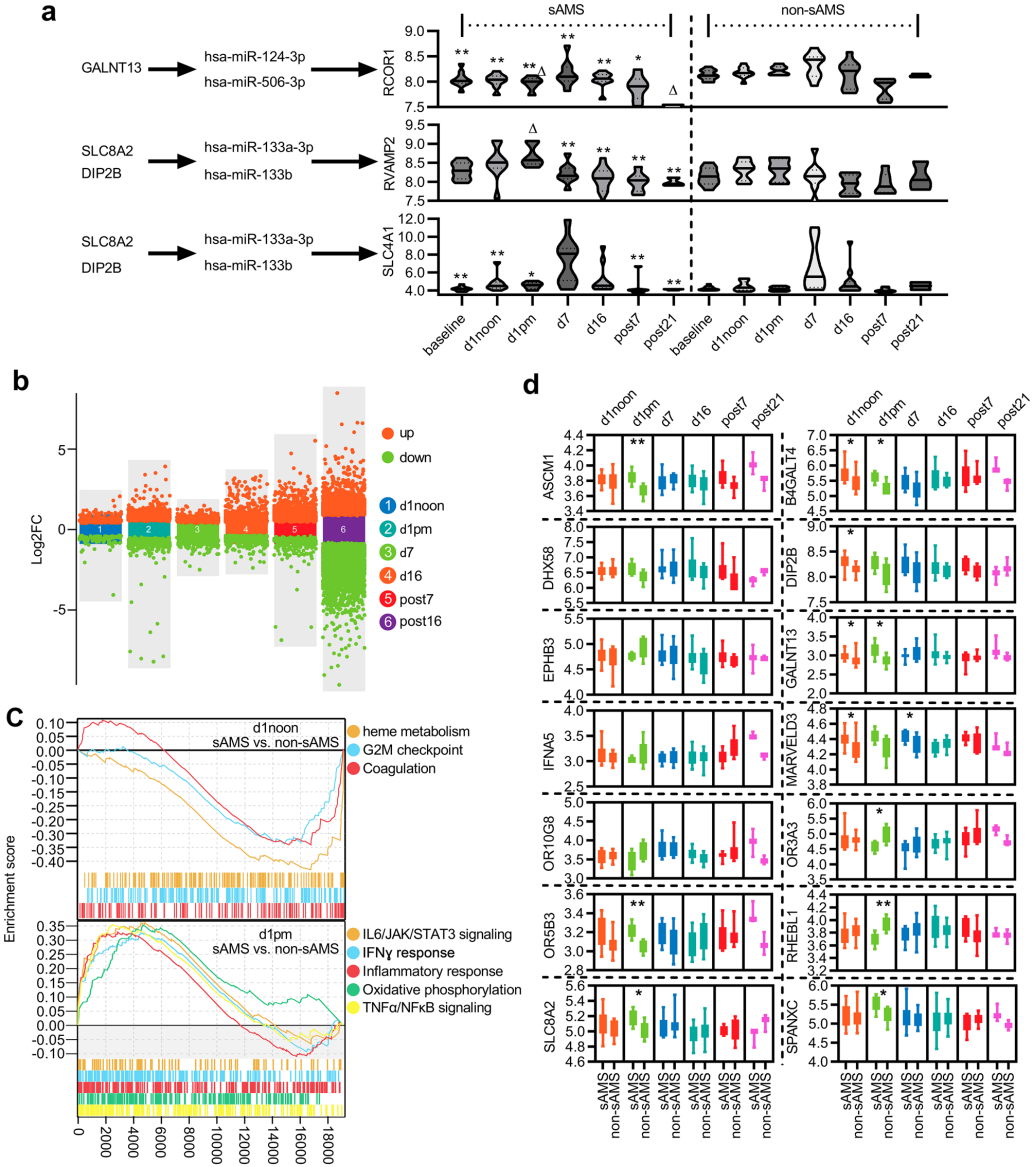
Changes of the featured genes and their miR targets in sAMS. (**a**) Timeline changes of miR targets. The miR products hsa-miR-124-3p/506-3p of GALNT13 mediated the expression changes in their target RCOR1 in sAMS, which peaked at d7 (N = 9) and recovered at post21 (N = 3). Compared with post21, *P < 0.05, **P < 0.01; in comparison with non-sAMS, ^∆^*P* < 0.05 (Tukey's multiple comparisons test). As the shared miR products of SLC8A2 and DIP2B, hsa-miR-133a-3p/133bregulate the expression of their targets, RVAMP2 and SLC4A1, which were upregulated at d1pm (N = 6) or d7 (N = 9). *P < 0.05, **P < 0.01 vs. d1pm or d7. ^∆^*P* < 0.05 vs. non-sAMS (Tukey's multiple comparisons test). (**b**) Between-group changes of miR targets. (**c**) GSEA analysis. (**d**) Between-group changes of the 14 featured genes. The featured genes were compared in the relative expression level between subjects with (N = 9 (d1noon), 6 (d1pm), 9 (d7), 10 (d16), 7 (post7), 3 (post21)) and without sAMS (N = 10 (d1noon), 6 (d1pm), 9 (d7), 11 (d16), 7 (post7), 3 (post21)). Compared with non-sAMS, *P < 0.05, **P < 0.01 (Wilcox-test).

**Figure 8:**
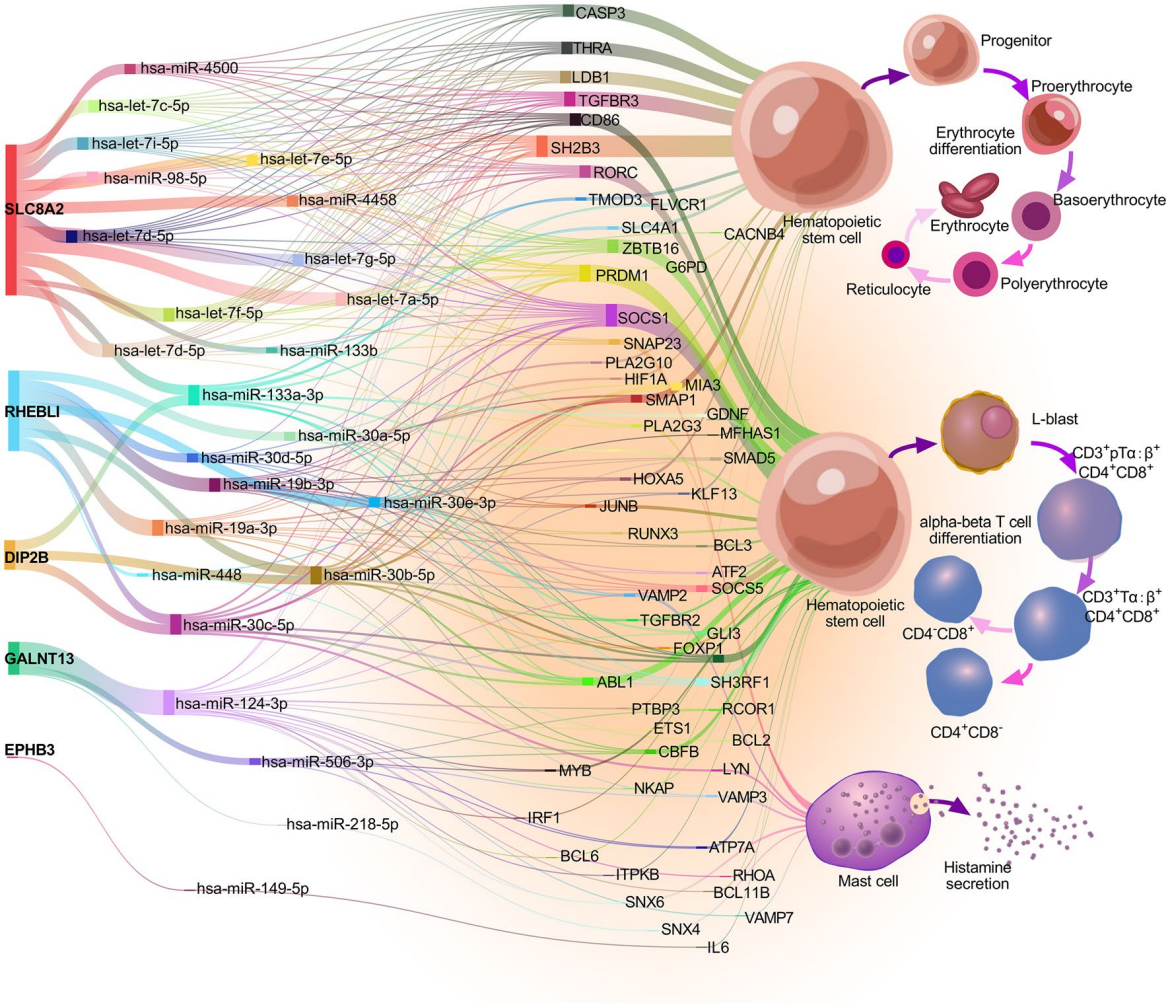
The identified pathways related to erythrocyte differentiation, alpha-bata T cell differentiation, and histamine secretion by mast cells.

There are 29 homo sapiens (hsa)-miRs identified in five featured genes, including 2 from EPHB3, 3 from DIP2B, 8 from RHEBL1, 3 from GALNT13, and 13 from SLC8A2, which were targeted to 3710 miR targets (Supplementary Fig. S4, Supplementary Table S12). We next wanted to determine the biological functions of the miR targets. As expected, most targets were enriched in terms related to lymphocyte activities under GO analysis (73.69%) (Fig. 6). Furthermore, 5.26% were enriched in histamine secretion, 2.56% in erythrocyte differentiation, and 15.79% in the regulation of myeloid cell differentiation (Supplementary Table S13). Accordingly, several meaningful pathways of the featured genes were identified, including GALNT13-(hsa-miR-124-3p/506-3p)–RCOR1, SLC8A2/DIP2B*-*(hsa-miR-133a-3p/133b*)-*RVAMP2/SLC4A1 (Fig. 7a), RHEBL1-(hsa-miR-19a/b-3p)-HIF1A, and EPHB3-(hsa-miR-149-5p)-IL6.Under GO and gene set enrichment analysis (GSEA), different expression profiles of the miR targets were observed between people with and without sAMS along the timeline (Fig. 7b), with pathways related to heme metabolism (P < 0.001, normalized enrichment score (NES) = − 1.703), G2M checkpoint (P = 0.023, NES = − 1.341), and coagulation (P = 0.040, NES = − 1.328) significantly down-regulated in sAMS at d1noon, while with oxidative phosphorylation (P < 0.001, NES = 1.624), inflammatory response (P < 0.001, NES = 1.543), IL6/JAK/STAT3 signaling (P = 0.012, NES = 1.492), IFNɣ response (P = 0.007, NES = 1.516), TNFα/NFκB signaling (P < 0.001, NES = 1.661) significantly up-regulated at d1pm, when sAMS occurred in 10 of 21 subjects observed (Fig. 7c). Furthermore, most of the 14 featured genes were observed with different expressions in sAMS vs. non-sAMS at d1noon, d1pm and/or d7 (Fig. 7d). All the featured genes and their miR targets were functionally related to erythrocyte differentiation, alpha–beta T cell differentiation, and histamine secretion by mast cells (Fig. 8). These results suggested the important roles of the featured genes in sAMS, which were mediated by miRs and their downstream targets.

## Discussion

The study was based on the microarray dataset abstracted from GSE103927^21^, a well-established dataset including 112 PBMC samples from 21 subjects who were rappidly exposed or re-exposed to the very high altitude of 5260 m after multiple periods of hypoxia acclimatization varying from 48 h to 21 days. Data at baseline, d1pm, d7, d16, and post7 from 21 subjects who completed all the planned tests were extracted for further analysis. On the first day of exposure, 10 of 21 were diagnosed with sAMS (LLQ-AMS score ≥ 6), a severe condition implying the possibility of life-threatening events. Although all of them recovered from a 3-night acclimatization at 3800 m followed by a prolonged stay at 5260 m for 13 days (d16), we still wondered why some of them were at risk of sAMS, but others were not, even under almost the same exposure conditions. In this study, the genetic basis underlying the pathological and physiological responses to VLH exposure was investigated to identify those who are vulnerable to sAMS or related events.

The gene expression patterns at d1pm and d7 varied from baseline but recovered after several acclimatization days at d16 and post7 (Fig. 1b), suggesting genetic responses upon acute VLH exposure. Most DGs across baseline, d1, and d7 (Fig. 1c) were involved in immune cell activation (Fig. 1d), with a continuous upregulation from baseline to the peak of d7 in those related to leukocyte activation upon immune response (Fig. 1e). Similar patterns of immune activation triggered by high-altitude exposure (3232 m) were also observed in another study^28^, with immune responses sensitized at the early phase of high-altitude exposure. Furthermore, peak changes in clustered DGs were observed at d7 (Fig. 2a), with functions related to T cells (gamma delta, CD8, CD4 naive, CD4 memory resting, and CD4 memory activated) dominating in DG clusters (Fig. 2b). Accordingly, these up- or downregulated DGs were functionally related to platelet activation, oxidative stress, and/or T cell differentiation (Fig. 2c), which are considered essential to the occurrence of AMS, the subsequent development of sAMS, and/or related events^29–33^. These results implied that T cells dominated the genetic responses to VLH exposure.

The proportion of CD4 T cells was indicated by a significant decrease in the subjects from baseline to d7 (Fig. 3a) and the inverted CD4/CD8 ratio (Fig. 3b), which was also reported in other high-altitude populations^33^, reminding us of the enhanced immunity and susceptibility to sAMS. Interestingly, similar timeline changes were observed in the CD4/CD8 ratio, LLQ-AMS score, and other laboratory values detected, all with peaked values at d1pm or d7, then recovered from d7 to d16 (Supplementary Fig. S5), implying their contributions to sAMS as risk factors. Then, the CD4/CD8 ratio, SaO_2_, CaO_2_, P50, hemoglobulin, and the estimated proportion of T cells were further investigated for the interplay effects between them, aiming to identify the underlying risk genes of sAMS (Fig. 3c,d). A total of 2286 risk genes were mapped (Fig. 4) for classifier training, and 14 gene classifiers were identified to establish the model using SVM-RFE (Fig. 5a–d). We established a ten-gene model of genetic susceptibility to sAMS (Fig. 5e) with excellent discrimination (C-index = 1, AUC = 1) and satisfactory predictive accuracy as assessed using ROC and survival analysis (Fig. 5f,g). We also constructed one-gene and three-gene models. All the models were indicated to have good clinical applicability as assessed by the overall net benefits over risks (Fig. 5h), suggesting the roles of the modeled genes as predictive markers for sAMS.

Limited evidence has indicated that certain miRs may function as biomarkers for AMS^34,35^ or play roles in acute hypoxia and hypoxia-induced pulmonary vascular leakage^36^. In subjects exposed to a height of 3100 m, miR-424 was overexpressed in a HIF1A-dependent manner, which in turn can stabilize HIF1A. In our study, 29 miRs and 3710 miR targets were identified from five genes (EPHB3, DIP2B, RHEBL1, GALNT13, and SLC8A2) (Supplementary Fig. S4), which were associated with multiple biological processes, as evidenced by GO analysis (Fig. 6). We identified 260 important miR-mediated signalling pathways concerning erythrocyte differentiation, alpha–beta T cell differentiation, and histamine secretion (Fig. 8). As one of the sodium-calcium exchangers, SLC8A2 has been previously shown to be a nuclear translocation regulator of HIF1A^37^, which was significantly downregulated upon SLC8A2 overexpression^38^. Our study indicated that SLC8A2 acts upstream of multiple hypoxia- and/or altitude-sensitive miR targets, such as RCOR1 (a transcription rheostat essential for normal myeloerythroid lineage differentiation)^39,40^, PRDM1 and LDB1 (both are involved in high-altitude adaptation)^41,42^, and CASP3 (a member of the hypoxia-activated mitochondrial apoptosis pathway)^43^. We noticed that both hsa-miR-133a-3p and hsa-miR-133b-3p mediate the signals from SLC8A2 or DIP2B to SLC4A1, a biomarker of AMS, which was correlated with various AMS symptoms and plays important roles in CO_2_ gas transport in erythrocytes^44^. The shared miR targets of SLC8A2 and DIP2B also include TMOD3, which has been shown to be a candidate biomarker for high-altitude pulmonary hypertension in Kyrgyz highlanders^45^. Interestingly, GALNT13 has been previously identified as a risk gene relevant to sickle cell disease-associated pulmonary hypertension, which may play roles in endothelial permeability^46,47^. Our results showed that GALNT13 interacts with multiple miR targets related to histamine secretion and hypoxia-induced activities in erythrocytes and T cells, suggesting its potential effects in pulmonary vascular and ventricular injuries. More importantly, as miR products of RHEBL1 (a member of Ras superfamily)^48^, both hsa-miR-19a-3p and hsa-miR-19b-3p were indicated as mediators of HIF1A^49,50^, suggesting important roles in hypoxia-related biological processes. Furthermore, the EPHB3 (a proliferation suppressor in ambient and hypoxic environments)^51^-(hsa-miR-149-5p)-IL6 pathway was also believed to be essential for hypoxic responses as the underlying association between hypoxia and inflammation. We failed to identify miR products or miR-mediated signals related to the other 9 featured genes, whereas some of them were previously argued to be hypoxia-sensitive genes, such as ACSM1, a member of the lipoic acid salvage pathway controlling HIF1 activation^52,53^. Obviously, as potential predictors or biomarkers of sAMS, the 14 featured genes still remain far from being uncovered regarding their roles and mechanisms in the development of sAMS.

Because of the infeasibility, it is almost impossible to conduct large-scale trials at high altitudes, especially under extreme conditions such as VLH. In our study, data of very small number of cases were used, which may result in biased machine learning performance. SVM seems good at dealing with small samples and large numbers of features, but the use of CV methods seems not sufficient to control overfitting^54^. Furthermore, higher dimensional interactions should be considered in machine learning, as it is possible that linear kernels based SVMs may not capture all non-linearity inherently. Though, in our study, completely separating validating and training data were applied and satisfactory prediction accuracy of the model was abtained, other model selection strategies may have superiority over SVM, like StepSVM^55^, Random Forest^56^, and Xgboost^57^. Fortunately, part of the selection results were repeated when using bagging-based Random Forest, and boosting-based Xgboost instead of SVM (Supplementary Fig. S6), suggesting the rationality of SVM-RFE strategy applied in sAMS prediction. We have explored the interplay effects between the laboratory values and LLQ-AMS score at the genetic level. It was indicated that SaO_2_ (AUC = 0.617), CaO_2_ (AUC = 0.783), P50 (AUC = 0.667), hemogbin (AUC = 0.850) functioned as possible binary classifiers for sAMS, suggesting their roles in determining sAMS. However, for inconsistent testing conditions and findings, these laboratory values may still be not available in the prediction of sAMS for prevention purposes.

This study was based on microarray data from 112 PBMC samples of 21 subjects exposed to VLH and aimed to explore the genetic susceptibility of sAMS. Using the machine learning of SVM-RFE, we identified 14 classifier genes and established a prediction model of sAMS, which performed well in predicting or differentiating subjects suffering from sAMS, and hold promise to be clinically applied in the early screening for sAMS risks. However, more studies are still needed to corroborate the existing findings related to the predictive or differentiating power of the model and to establish the role of the modeled genes as biomarkers for sAMS.

## Methods

### Collection and preprocessing of VLH microarray data

VLH microarray data were explored in Gene Expression Omnibus^58^ using the keywords “AMS” and “high altitude” and were collected from the platform GPL6244 in MINiML format under the accession numbers GSE103927 and GSE52209. GSE103927 was structured on 112 PBMC samples from 21 subjects exposed to sea level then VLH at seven time points, including baseline, the first day noon (d1noon) or post meridiem (d1pm), day 7 (d7), day 16 (d16), post7 or post21. Information concerning age, sex, height, detected levels of PaO_2_, PaCO_2_, SaO_2_, CaO_2_, P50, hemoglobin, LLQ-AMS score, and AMS-C-Composite score were included (Supplementary Table S14). Data of d1 were used to train and establish the model. Data from baseline, d7, d16, and post7 were used to test the model. Isolated data from GSE52209 were applied to validate the model. All the raw data were extracted and preprocessed using the package in RStudio (2022.07.1 + 554) and then normalized by log2 transformation using the normalize quantiles function of the preprocessCore package. The normalized data were annotated in GPL6244 for the conversion of all probes into gene symbols. Probes mapping to multiple genes were filtered out. The final gene expression value was determined by the mean over multiple detection. The removeBatchEffect function of the limma package was applied to remove the batch effects. PCA was performed prior to further analysis of all the data, to determine whether gene expression changes over time or whether timeline datasets depend on one another. No live human/ blood sample were used for the study.

### Identification of DGs

DGs across baseline, d1pm and d7 in the training cohort were identified using the Limma package (version: 3.52.3) in R software. False positive results were corrected via P-value adjustment. The thresholds for the screening of DG mRNAs were defined as *P* < 0.05 and log2FC > 1.3 or log2FC <  − 1.3.

### Function and pathway enrichment

GO analysis, GSEA and their visualization were performed using the ClueGo application in Cytoscape software (version 3.9.1)^59^. Various types of evidence (experimental, computational, author statement from publication, and curatorial statement) were used for analysis. Ontologies, pathways, and annotation files were updated before each analysis using the GO annotation database (UniProt-GOA)^60^. To identify the representative pathways, medium network specificity was selected, and GO levels varying from 3 to 8, with a minimum of 3 genes per term and at least 4% of the total associated genes, were mapped. The GO term fusion threshold was 50% for group merging. Only terms with a P value < 0.05 were displayed with statistical significance.

### Cluster analysis

DGs across baseline, d1pm, and d7 in the training cohort were explored for functional differentiation of leukocytes using fuzzy c-means clustering. The mean expression value of DGs at each time point was calculated using the avereps function in the limma package. The expression patterns along the exposure timeline were detected with the Mfuzz package. The filter threshold for the expression value was 0.25. According to the results of multiple rounds of training, the number of clusters was expected to be 6. Each cluster was compared in relation to immune signatures using GO analysis and LM22, a leukocyte gene signature matrix containing a total of 547 leukocyte markers^24^. Pearson’s test was used to estimate the timeline correlation of each cluster to LM22 signatures.

### Cibersort algorithm for the abundance of leukocyte types in PBMC samples

Coupled with LM22, the cibersort algorithm was used to distinguish 22 types of leukocytes, including B cells, plasma cells, T cells, NK cells, monocytes, macrophages, dendritic cells, mast cells, eosinophils, neutrophils, and the subtypes described above^25^. The timeline expression data from 112 samples were extracted for analysis. To improve the accuracy of the deconvolution algorithm, 1000 permutations from the default signature matrix were applied to compute the P value and root-mean-square deviation for samples at each time point. The scores for each signature were summarized and median centered to permit timeline comparisons. The paired T test was used to discover the significance between comparisons, with parametric test performed to assume Gaussian distribution. The total estimated sum of CD4 T cells (naive, memory resting, and memory activated) and CD8 T cells was used to calculate the CD4/CD8 ratio. All T cell subtypes were used to estimate the total proportion of T cells. ROC was used to predict the best thresholds of SaO_2_, P50, hemoglobin, CaO_2_, and estimated proportion of T cells, as binary classifiers of CD4/CD8 ratio, and sAMS (LLQ-AMS score ≥ 6).

### Risk gene mapping

The interplay effects between the estimated CD4/CD8 ratio, LLQ-AMS score, SaO_2_, P50, hemoglobin, CaO_2_, estimated proportion of T cells, and underlying hub genes were visualized using a nine-quadrant diagram and Venn diagram^26^. WGCNA^27^ was conducted to discover the relationships between gene expression patterns and phenotypes. Genes with expression values above 1 were applied for further analysis. The soft power was estimated at 7. An unsigned scale-free coexpression network for the genes was constructed using the minimum module size (minModuleSize) of 100 and the threshold of 0.25 for merging of modules. Pearson’s correlation value was applied to establish the similarity matrix, adjacency matrix, and topological overlap matrix between each pair of genes across all samples. The gene coexpression module was detected using the dynamic tree cut algorithm and was constructed with a cut height of 0.975.

### Machine learning to establish the prediction model of sAMS

The phenotype-validated DGs were input into the SVM-RFE system for machine learning, which was run within the e1071 and msvmRFE packages. SVM-RFE is an SVM-based iterative algorithm that works backward from an initial set of features and is applied to find the optimal hub gene by deleting feature vectors. The input data were from all samples of the training cohort of d1, containing 19 observations (individual subjects) and 2286 features (DGs). We used k = 15 for the k-fold CV and halve.above = 30 to cut the features in half each round until there were fewer than 30 remaining features. The entire feature selection and generalization error estimation process was wrapped for five-fold CV. Feature ranking was performed using the lapply function based on the average rank across the five folds of accuracy and error estimation. Univariate Cox hazard analysis was applied to assess the prediction performance of the selected features. Multinomial logistics regression, nomogram, survival analysis, AUC, and calibration curve were used to establish, test, and validate the model. DCA was used to assess the clinical applicability of the prediction model. The model was tested within the timeline of the training cohort, including baseline, d7, d16, and post7, and validated in another cohort, which comprised 17 subjects who developed high-altitude pulmonary edema within 48–72 h after exposure to VLH, 14 normal controls, and 14 high-altitude natives (GSE52209)^23^. R packages including Hmisc, lattice, survival, Formula, ggplot2, rmda, ggDCA, rms, SparseM, caret, and pROC were applied in the model development.

### MiR analysis

To further explore the roles of the 14 featured genes in the development of sAMS, miRs and their targets were predicted using the miR function of FunRich (version 3.1.3). Timeline changes in miR targets in sAMS were analysized using ordinary one-way analysis of variance. Tukey's multiple comparisons were performed to compare the mean expression of each targets with the mean value of other targets. Testing the homogeneity of variances (equality variances) among different time points was done using the Brown–Forsythe test. All the miR targets were compared in the expression profiles between sAMS and non-sAMS using GO and GSEA tools. The between-group changes of the featured genes were analyzed using Wilcox-test, with the hypothetical value set as zero. All the values matching zero in the datasets were entirely ignored in the analysis. All the methods used in the study were outlined in the flowchart (Supplementary Fig. S7).

## Supplementary Information

Supplementary Figures.

Supplementary Table S1.

Supplementary Table S2.

Supplementary Table S3.

Supplementary Table S4.

Supplementary Table S5.

Supplementary Table S6.

Supplementary Table S7.

Supplementary Table S8.

Supplementary Table S9.

Supplementary Table S10.

Supplementary Table S11.

Supplementary Table S12.

Supplementary Table S13.

Supplementary Table S14.

## Data Availability

Data sets described in this article are available from Gene Expression Omnibus (http://www.ncbi.nih.gov/geo) via the accession number GSE103927 and GSE52209. All data generated in this article are freely accessible in supplementary tables to any scientist wishing to use them noncommercially. On reasonable request, the corresponding author can provide further information.
