# Supplementary material for: Establishing a prediction model of severe acute mountain sickness using machine learning of support vector machine recursive feature elimination
Source: Sci Rep. 2023 Mar 21;13:4633. doi: 10.1038/s41598-023-31797-0 (PMC10030784; doi:10.1038/s41598-023-31797-0)
Supplement: Supplementary file 1 — Supplementary Figures. [file 41598_2023_31797_MOESM1_ESM.pdf]

Establishing a prediction model of severe acute mountain sickness using machine learning of support vector machine recursive feature elimination

Min Yang[1*], Yang Wu[1], Xing-biao Yang[1], Tao Liu[1], Ya Zhang[1], Yue Zhuo[1], Yong Luo[1], Nan Zhang[2]

[1] Department of Traditional Chinese Medicine, Rheumatology Center of Integrated Medicine, The General Hospital of Western Theater Command, PLA, Chengdu, 610083, China

[2.] Department of Hematology, The General Hospital of Western Theater Command, PLA, Chengdu, 610083, China

[*]Correspondence:

Email: translie@live.cn (M.Y.)

Supplementary Figures

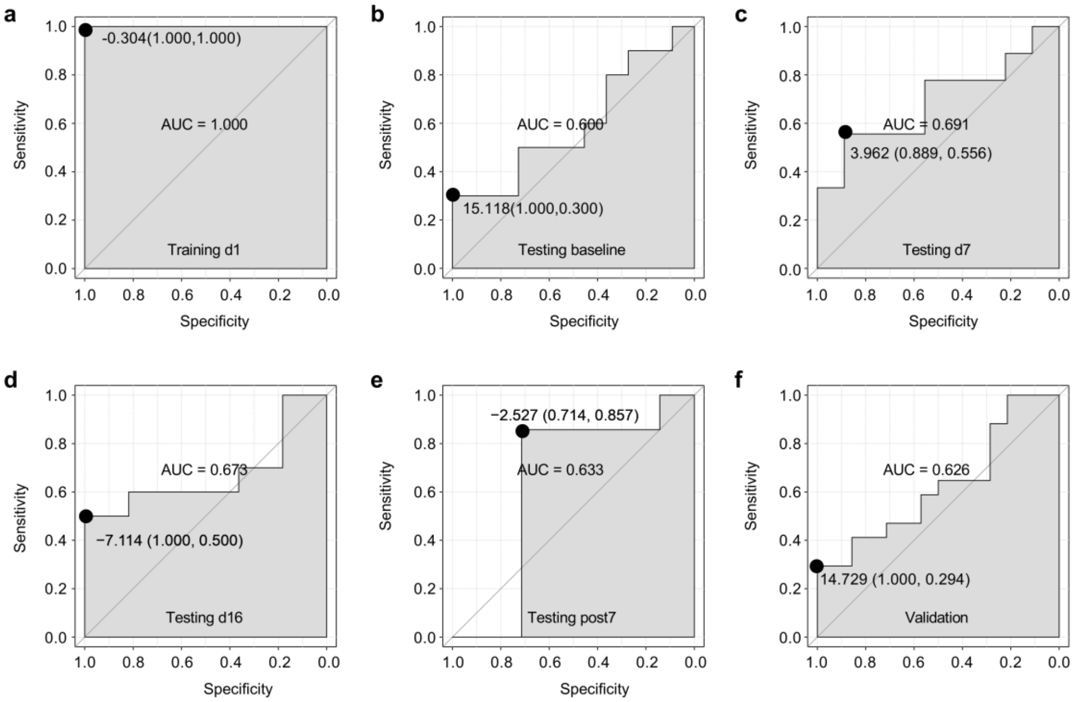

Fig. S1 Testing and valiation of the ten-gene model using ROC. **(a)** Self-testing using the training-cohort data of d1. (**b)** Self-testing using the training-cohort data of baseline. (**c)** Self-testing using the training-cohort data of d7. (**d)** Self-testing using the training-cohort data of d16. (**e)** Self-testing using the training-cohort data of post7. (**f)** Valiation of the model using the data of the validation cohort. The threshold point is displayed with the black dot. AUC, area under curve; d1, day 1; post7, post-decent day 7; ROC, receiver operating characteristic curve.

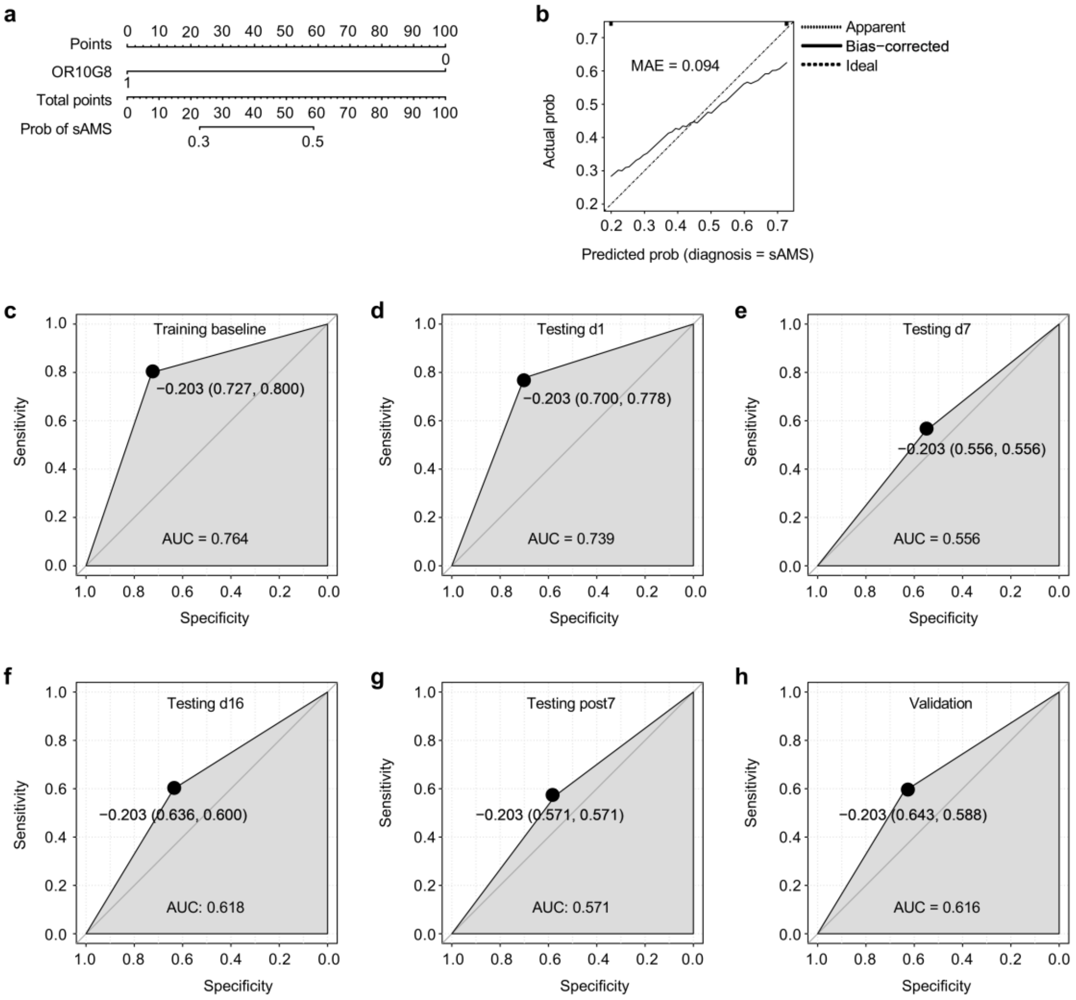

Fig.S2 Construction, testing and validation of the one-gene model. (**a**) Nomogram of the model. (**b**) Calibration performance of the model. (**c**) Self-testing using the training-cohort data of baseline. (**d**) Self-testing using the training-cohort data of d1. (**e**) Self-testing using the training-cohort data of d7. (**f**) Self-testing using the training-cohort data of d16. (**g**) Self-testing using the training-cohort data of post7. (**h**) Valiation of the model using the data of the validation cohort. The threshold point is displayed with the black dot. AUC, area under curve; d1, day 1; MAE, mean absolute error; post7, post-decent day 7; prob, probability; sAMS, severe acute mountain sickness.

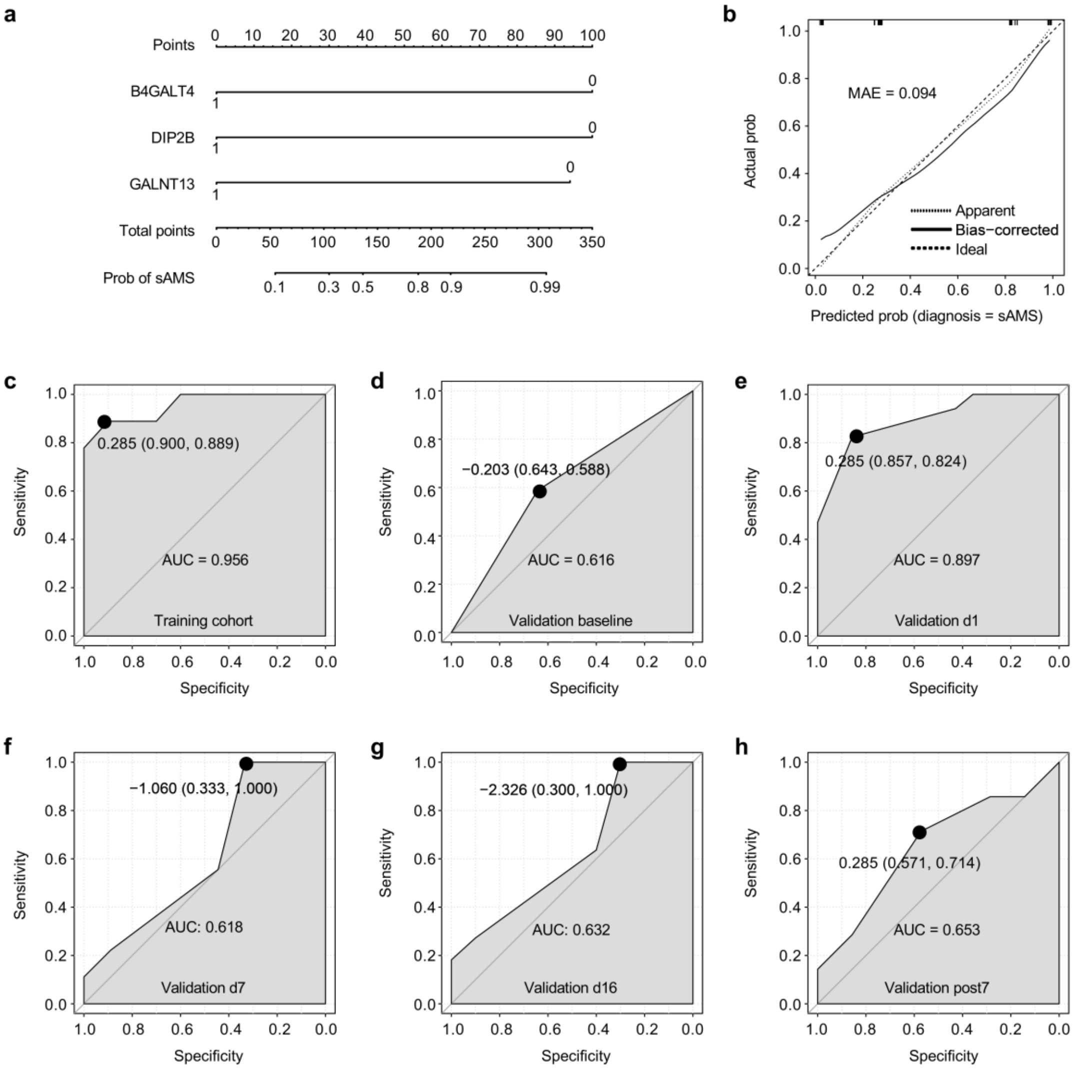

Fig.S3 Construction, testing, and validation of the three-gene model. **(a)** Nomogram of the model. **(b)** Calibration performance of the model. **(c)** Self-testing using the validation-cohort data. **(d)** Validation using the training-cohort data of baseline. **(e)** Validation using the training-cohort data of d1. **(f)** Validation using the training-cohort data of d7. **(g)** Validation using the training-cohort data of d16. **(h)** Valiation using the the training-cohort data of post7. The threshold point is displayed with the black dot. AUC, area under curve; d1, day 1; MAE, mean absolute error; post7, post-decent day 7; prob, probability; sAMS, severe acute mountain sickness.

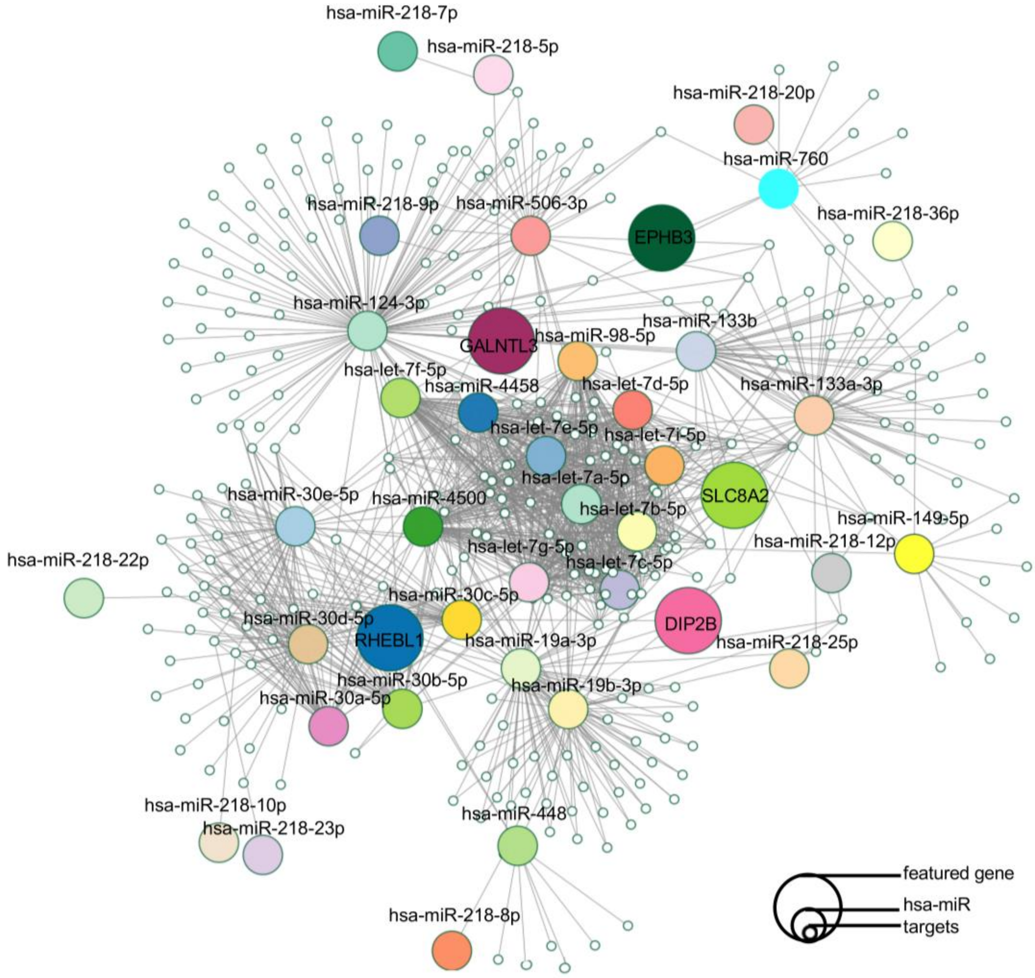

Fig. S4 The featured gene-miR-target network.

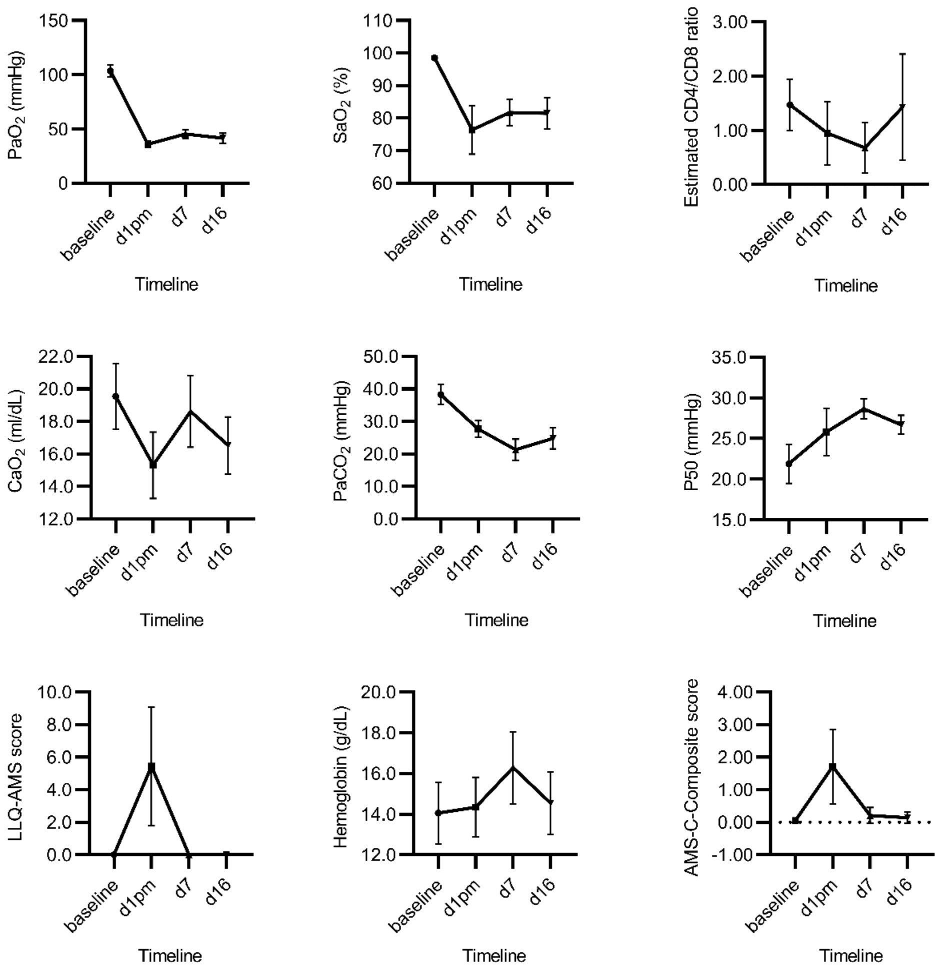

Fig. S5 The timeline changes of the potential risk factors for sAMS. The error bars indicated 95% CI. Abbreviations: AMS, acute mountain sickness; CaO_2, arterial oxygen content; CI, confidence interval; d1noon, day 1 noon; d1pm, day 1 post meridiem; LLQ, Lake Louise Questionnaire; PaO_2, partial pressure of oxygen; $PaCO_2$, partial pressure of carbon dioxide; post7, post-decent day 7; SaO_2, saturation of oxygen; P50, oxygen tension at 50% hemoglobin saturation.

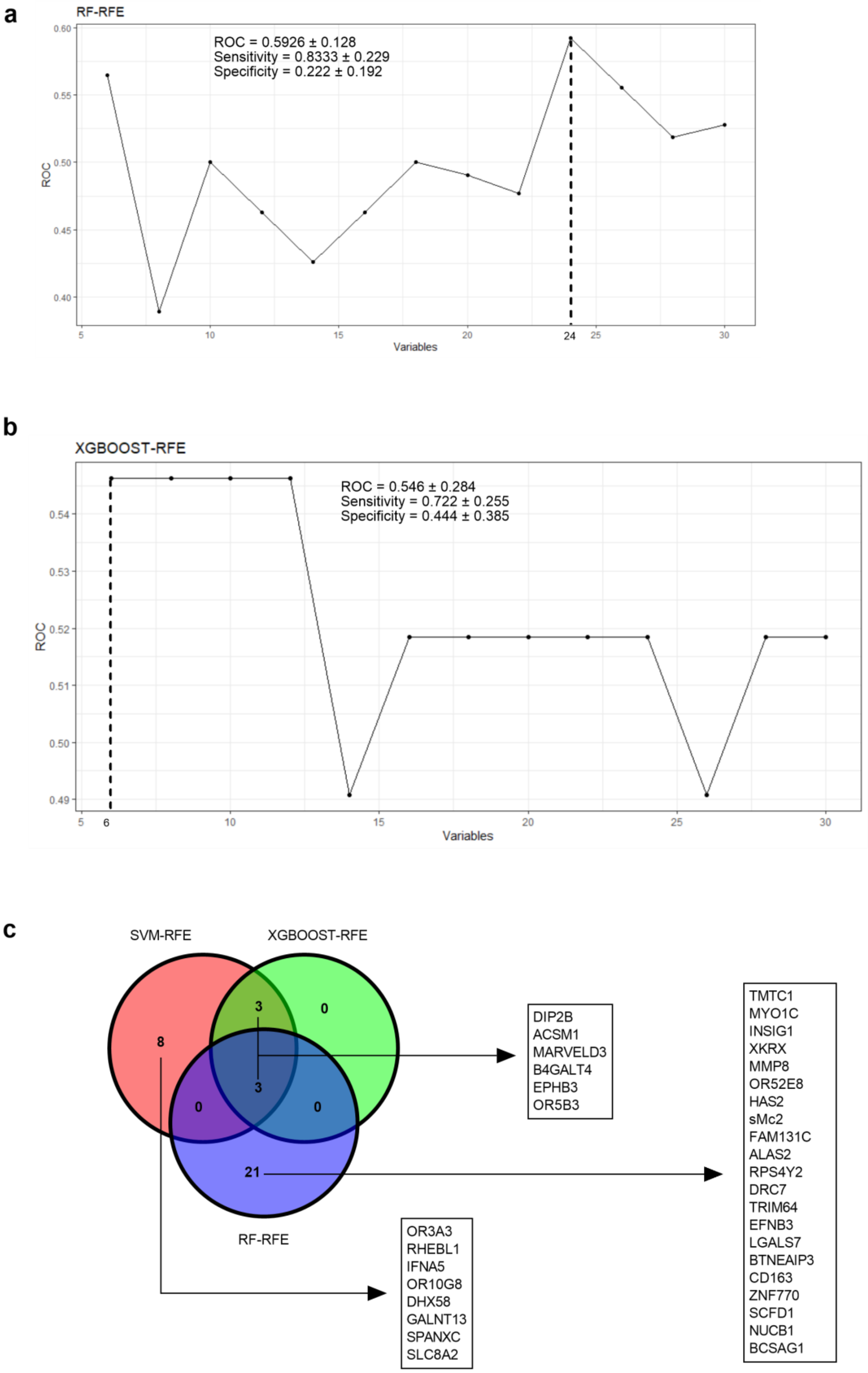

Fig.S6 Features selected using RF-RFE and XGBOOST-RFE**. (a)** Best AUC when 24 featured genes

were selected using RF-RFE. **(b)** Best AUC when 6 featured genes were selected using XGBOOST-RFE. **(c)** Intersected features selected using SVM-RFE, RF-RFE, and XGBOOST-RFE. Abbreviations: AUC, area under curve; RFE, recursive feature elimination; RF, Random Forest; ROC, receiver operating characteristic curve; XGBOOST, extreme gradient boosting. Both RF-RFE and XGBOOST-RFE were performed using R packages including caret, tidyverse and readxl.

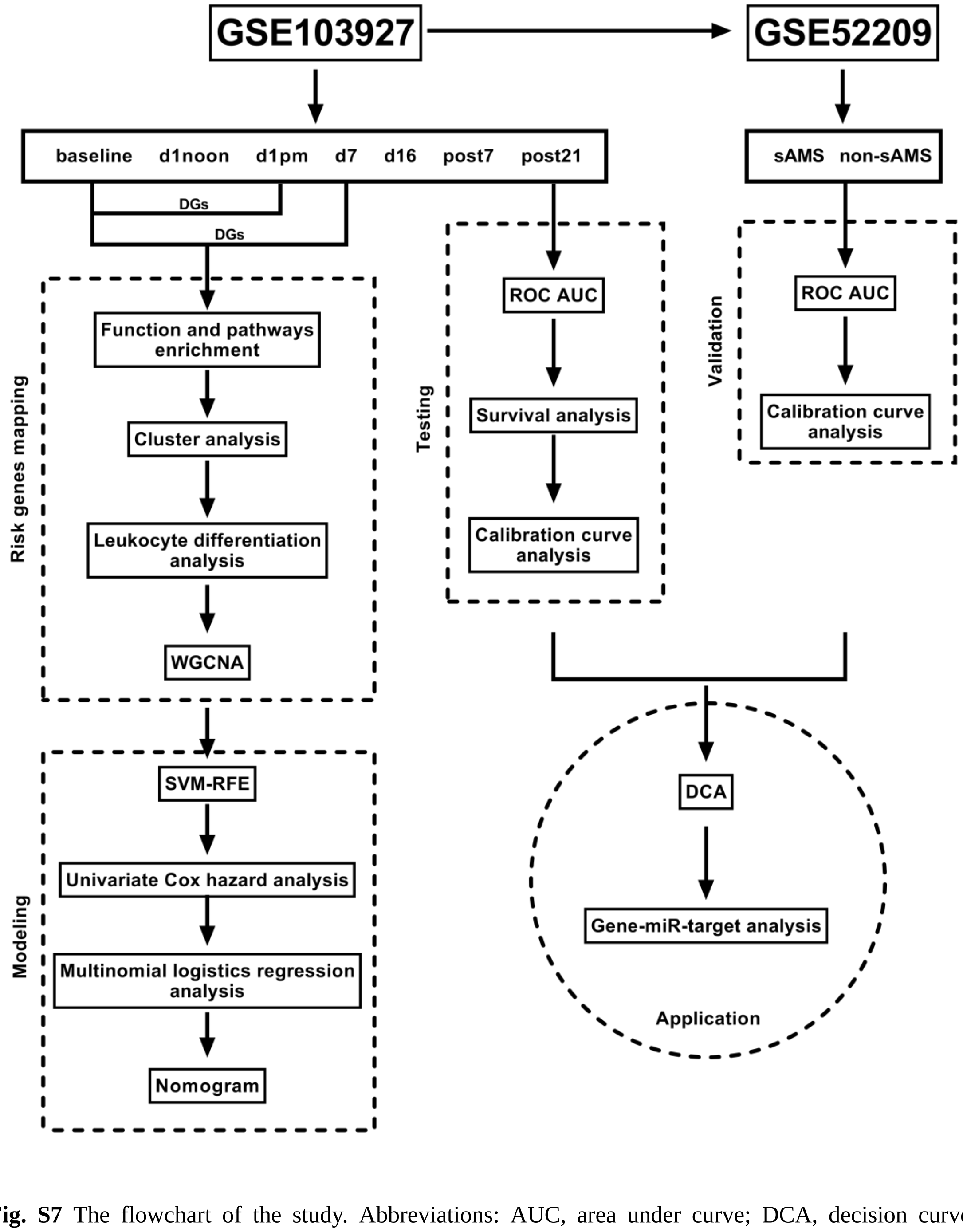

Fig. S7 The flowchart of the study. Abbreviations: AUC, area under curve; DCA, decision curve analysis; d1noon, day 1 noon; d1pm, day 1 post meridiem; post7, post-decent day 7; DGs: differentially expressed genes; ROC, receiver operating characteristic curve; sAMS, severe acute mountain sickness; SVM-RFE, support vector machine recursive feature elimination; WGCNA, weighted correlation network analysis.
